# Health-Related Quality of Life in the US Territories of Puerto Rico, Guam, and the Virgin Islands

**DOI:** 10.1001/jamanetworkopen.2025.5646

**Published:** 2025-04-17

**Authors:** Anna-Michelle Marie McSorley, Adrian Matias Bacong

**Affiliations:** 1 Department of Allied Health Sciences, College of Agriculture, Health and Natural Resources, University of Connecticut, Waterbury; 2 Center for Anti-Racism, Social Justice, and Public Health, School of Global Public Health, New York University, New York; 3 Center for Asian Health Research and Education, School of Medicine, Stanford University, Stanford, California; 4 Department of Medicine, Division of Cardiovascular Medicine, School of Medicine, Stanford University, Stanford, California

## Abstract

This cross-sectional study examines the mental and physical health-related quality of life in US territories compared with the 50 US states.

## Introduction

Although part of the US, the territories of Puerto Rico, Guam, and the Virgin Islands are regularly excluded in studies examining national health trends, often due to limited data availability.^1,2^ Thus, empirical reports on population characteristics and health status of US territories remain scant. In this cross-sectional study, we examine mental and physical health-related quality of life in 3 territories compared with the 50 states, collectively.

## Methods

Our cross-sectional study adhered to the Strengthening the Reporting of Observational Studies in Epidemiology (STROBE) reporting guideline. Our study did not include human participants and was exempt from institutional review board at Stanford University. Additional covariate and methodological details are in eMethods in Supplement 1.

We conducted a secondary quantitative analysis of pooled data from the 2021 and 2022 Behavioral Risk Factor Surveillance System (BRFSS), a probability-based survey of noninstitutionalized US residents in the 50 states, Puerto Rico, Guam, and US Virgin Islands.^3^ The BRFSS collects self-reported data from more than 400 000 adults annually through random cellular phone and landline calls. We included all participants aged 18 years or older in our jurisdictions of interest who had complete data for our health outcomes. Our study sample represented 240 527 886 adults after applying survey weights. To assess population-level physical and mental health, we used 4 self-rated and validated health-related quality of life items (HRQOL-4): general health (Likert-type scale), physical health (measured in 0 to 30 days), mental health (measured in 0 to 30 days), and activity limitations (measured in 0 to 30 days).^4^ Using Stata version 17.0 (StataCorp), we examined differences in sociodemographic factors, age-adjusted prevalence, and fully-adjusted prevalence for HRQOL-4 items across 3 US territories compared with the 50 states. Data were analyzed between December 2023 and January 2025.

## Results 

Our study sample included 830 390 individuals (Puerto Rico, n = 9280 [1.1%]; Guam, n = 3793 [0.5%]; US Virgin Islands, n = 2642 [0.3%]; all 50 states, n = 814 675 [98.1%]), with notable differences in sociodemographic compositions (Table). Analyses found noticeable heterogeneity for the age-adjusted and fully-adjusted prevalence of 4 quality of life measures (Figure) for the territories compared with the 50 states. The results reported represent age-adjusted values. Higher proportions of individuals in Puerto Rico (27.8%; 95% CI, 26.6%-29.0%), US Virgin Islands (18.6%; 95% CI,14.0%-23.2%), and Guam (17.7%; 95% CI, 15.2%-20.2%) reported fair or poor general health compared with the 50 states (16.1%; 95% CI, 16.0%-16.3%). For poor physical health, people in Puerto Rico had higher proportions (16.0%; 95% CI, 15.0%-17.0%), followed by US Virgin Islands (12.4%; 95% CI, 7.5%-17.4%), relative to the 50 states (11.5%; 95% CI, 11.3%-11.6%). However, proportions in Guam (10.7%; 95% CI, 8.6%-12.8%) were approximating those of the 50 states. For mental health, Puerto Rico (12.8%; 95% CI, 11.8%-13.7%), Guam (12.4%; 95% CI, 10.1%-14.7%), and US Virgin Islands (11.7%; 95% CI, 7.3%-16.1%) had lower levels of poor mental health than the 50 states (14.9%; 95% CI, 14.8%-15.1%). Finally, Puerto Rico (7.8%; 95% CI, 7.0%-8.6%) had similar proportions of activity limitations when compared with the 50 states (8.5%; 95% CI, 8.4%-8.7%); whereas Guam (6.9%; 95% CI, 5.1%-8.8%) and US Virgin Islands (7.0%; 95% CI, 3.5%-10.4%) values were slightly lower. Across all outcomes, in all jurisdictions, results remain similar in the fully-adjusted prevalence; however, Puerto Rico had the greatest health disparities relative to the other jurisdictions (Figure).

**Table.** Weighted Sample Characteristics for 50 States, Puerto Rico, Guam, and the US Virgin Islands, Behavioral Risk Factor Surveillance System, 2021 to 2022

Characteristics	Weighted % (95% CI)			
	All 50 states (n = 814 675)	Puerto Rico (n = 9280)	Guam (n = 3793)	US Virgin Islands (n = 2642)
Age, y				
18-39	37.3 (37.0-37.5)	37.4 (36.0-38.9)	44.1 (41.2-47.1)	30.6 (26.7-34.9)
40-64	40.6 (40.4-40.9)	39.0 (37.6-40.3)	43.5 (40.5-46.5)	48.9 (43.8-54.1)
≥65	22.1 (21.9-22.3)	23.6 (22.5-24.7)	12.4 (10.5-14.5)	20.4 (17.0-24.4)
Sex				
Female	51.2 (51.0-51.4)	53.0 (51.5-54.5)	49.5 (46.4-52.5)	51.4 (46.3-56.3)
Male	48.8 (48.6-49.0)	47.0 (45.5-48.5)	50.5 (47.5-53.5)	48.6 (43.4-53.7)
Education				
Less than high school	11.1 (11.0-11.3)	20.5 (19.2-21.9)	16.3 (13.6-19.2)	25.0 (20.3-30.3)
High school or GED	27.4 (27.1-27.6)	27.8 (26.5-29.1)	36.7 (33.9-39.6)	35.4 (30.5-40.5)
Some college or technical school	30.6 (30.3-30.8)	22.1 (21.0-23.3)	22.3 (20.0-24.8)	17.5 (14.0-21.7)
College graduate or above	30.9 (30.7-31.1)	29.6 (28.4-30.8)	24.8 (22.4-27.3)	22.2 (18.9-25.8)
Income				
<$15 000	5.2 (5.0-5.3)	28.5 (27.2-29.9)	10.2 (8.2-12.5)	11.9 (8.1-17.2)
$15 000-$24 999	7.8 (7.7-8.0)	22.2 (21.0-23.4)	12.2 (10.3-14.4)	11.1 (8.5-14.3)
$25 000-$34 999	9.6 (9.5-9.8)	10.0 (9.2-10.8)	11.3 (9.6-13.4)	14.3 (10.5-19.1)
$35 000-$49 999	10.1 (9.9-10.2)	8.6 (7.9-9.5)	13.0 (11.2-15.0)	11.2 (8.5-14.5)
≥$50 000	45.4 (45.2-45.7)	9.7 (8.9-10.5)	33.8 (31.2-36.6)	26.5 (22.9-30.4)
Missing	21.9 (21.7-22.1)	21.0 (19.8-22.2)	19.5 (16.9-22.3)	25.1 (20.9-29.8)
Insurance status				
No insurance	8.1 (7.9-8.2)	4.1 (3.5-4.8)	13.4 (11.4-15.7)	18.6 (14.8-23.2)
Has insurance	87.4 (87.2-87.6)	95.0 (94.3-95.7)	84.8 (82.4-86.9)	77.4 (72.7-81.5)
Missing	4.5 (4.4-4.6)	0.8 (0.6-1.2)	1.8 (1.1-2.8)	4.0 (2.6-6.2)

Abbreviation: GED, General Education Development Test.

**Figure.**
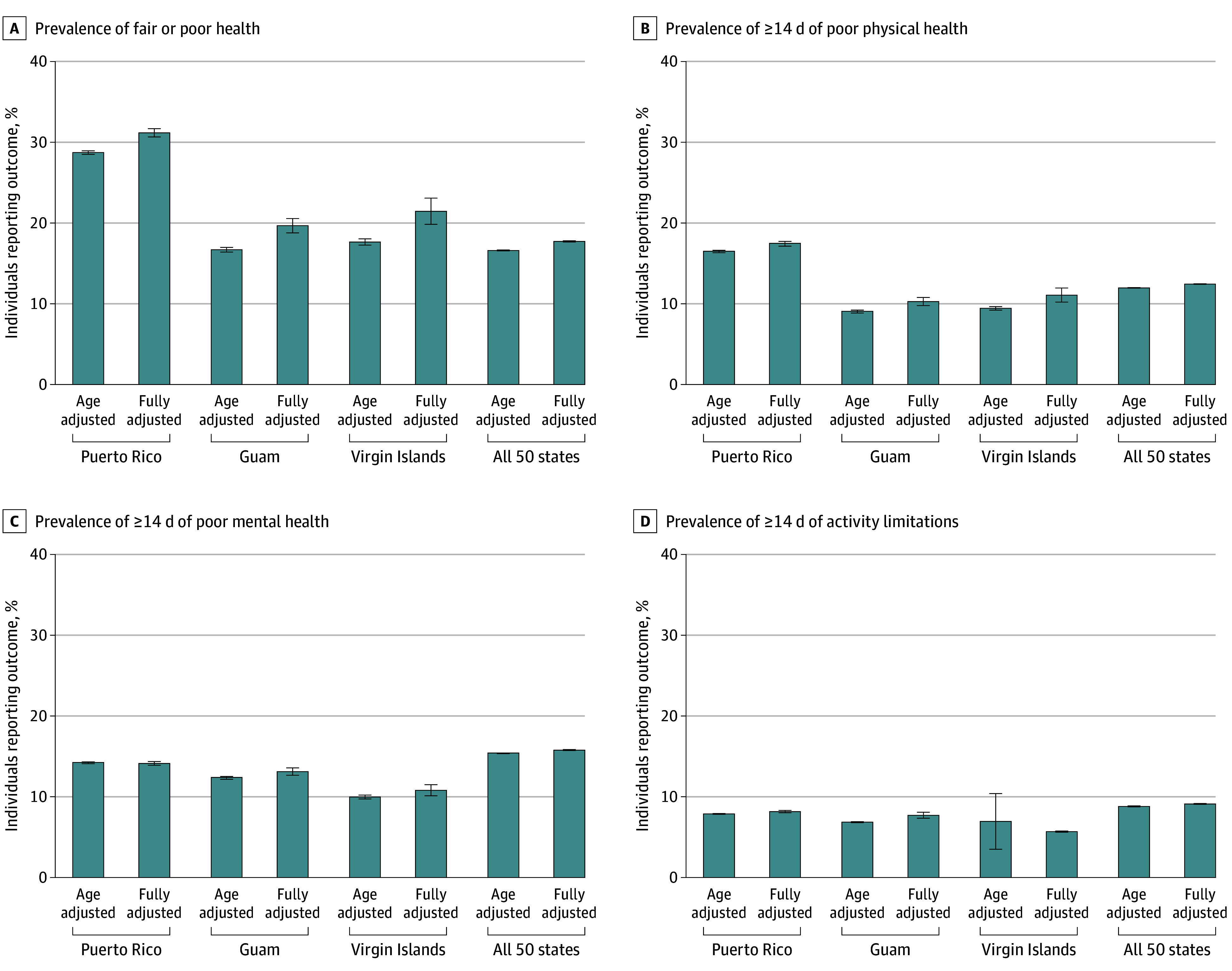
Adjusted Health-Related Quality of Life Outcomes for 3 US Territories and 50 states, Behavioral Risk Factor Surveillance System 2021 to 2022 Error bars indicate 95% CIs.

## Discussion

We found disparities in physical and mental health among territorial populations compared with the 50 states. Our findings in Puerto Rico, where more than one-quarter of respondents reported fair or poor health, partly contextualize the unexpectedly high number of deaths in Puerto Rico in 2022.^5^ Conversely, the territories had better mental health outcomes than the 50 states. However, levels of poor mental health were alarmingly high across all jurisdictions. While our findings do not have causal implications and are limited by self-report, they form an observational evidence-base that supports calls for mental health research or service expansion in the US and territories.^6^ However, gaps in federal health data systems (eg, Northern Mariana Islands and American Samoa are not in BRFSS)^1^ will thwart future efforts to provide this necessary evidence-base if allowed to persist.

## Notes

Supplement 1.
eMethods.

Supplement 2.
Data Sharing Statement

## References

[1] McSorley AM, Wheatley A, Pagán JA. A call to increase health data availability in US territories—not too small to count. JAMA Health Forum. 2023;4(9):e233088. doi:10.1001/jamahealthforum.2023.3088 37738063

[2] McSorley AM, Cui B, Kim J, Kuhn R. Gaps in US public health monitoring and surveillance systems in Puerto Rico. Am J Prev Med. 2024;66(3):551-558. doi:10.1016/j.amepre.2023.11.00337931723

[3] BRFSS overview CDC. US Centers for Disease Control and Prevention. Accessed January 12, 2024. https://www.cdc.gov/brfss/annual_data/2022/pdf/Overview_2022-508.pdf

[4] 2022 BRFSS questionnaire. US Centers for Disease Control and Prevention. Accessed January 12, 2024. https://www.cdc.gov/brfss/questionnaires/pdf-ques/2022-BRFSS-Questionnaire-508.pdf

[5] Pascual OS, Wiscovitch J, Tran AB, Hernández AR, Moriarty D. More people are dying in Puerto Rico as its healthcare system crumbles. *The Washington Post*. Accessed July 29, 2024. https://www.washingtonpost.com/nation/interactive/2023/puerto-rico-deaths/

[6] White House shares government, private sector, academic, and nonprofit actions to accelerate progress on mental health research. The White House. Accessed July 29, 2024. https://www.whitehouse.gov/ostp/news-updates/2024/06/03/white-house-shares-government-private-sector-academic-and-non-profit-actions-to-accelerate-progress-on-mental-health-research/

