# Supplementary material for: Health-Related Quality of Life in the US Territories of Puerto Rico, Guam, and the Virgin Islands
Source: JAMA Netw Open. 2025 Apr 17;8(4):e255646. doi: 10.1001/jamanetworkopen.2025.5646 (PMC12006872; doi:10.1001/jamanetworkopen.2025.5646)
Supplement: Supplement 1. — eMethods. [file jamanetwopen-e255646-s001.pdf]

Supplemental Online Content

McSorley AM, Bacong AM. Health-related quality of life in the US territories of Puerto Rico, Guam, and the Virgin Islands. *JAMA Netw Open*. 2025;8(4):e255646.
doi:10.1001/jamanetworkopen.2025.5646

eMethods.

This supplemental material has been provided by the authors to give readers additional information about their work.

© 2025 McSorley A-MM et al. *JAMA Network Open*.

eMethods

Outcome Variables
We examine four health-related quality of life outcomes: self-rated general health, self-rated physical health, self-rated mental health, and self-reported activity limitations.

Self-rated general health was asked as the following: “Would you say that in general your health is …?” Seven choices were possible: Excellent, Very Good, Good, Fair, Poor, Don’t know/Not sure, and Refused. We coded individuals who indicated that their health was “Excellent”, “Very Good”, or “Good” as “0” while individuals who indicated that their health was “Fair or Poor” as “1”. Individuals who indicated that they “Don’t Know/Not Sure” or “Refused” were coded as missing.

Self-rated physical health was asked as the following: “Now thinking about your physical health, which includes physical illness and injury, for how many days during the past 30 days was your physical health not good?”. Individuals would then indicate the number of days (Range 1 to 30 days), zero days (coded as “None”), “Don’t know/Not sure” or “Refused”. We dichotomized self-rated physical health, coding individuals who indicated that they had zero days of poor physical health or 1 to 13 days of poor physical health as “0” while individuals who had 14 to 30 days of poor physical health as “1”. Individuals who indicated “Don’t know/Not sure” or “Refused” were coded as missing.

Self-rated mental health was asked as the following: “Now thinking about your mental health, which includes stress, depression, and problems with emotions, for how many days during the past 30 days was your mental health not good?” Like self-rated physical health, individuals could indicate the number of days (Range 1 to 30 days), zero days (coded as “None”), “Don’t know/Not sure” or “Refused”. We dichotomized self-rated mental health, coding individuals who indicated that they had zero days of poor mental health or 1 to 13 days of poor mental health as “0” while individuals who had 14 to 30 days of poor mental health as “1”. Individuals who indicated “Don’t know/Not sure” or “Refused” were coded as missing.

Finally, self-reported activity limitations were asked as the following: “During the past 30 days, for about how many days did poor physical or mental health keep you from doing your usual activities, such as self-care, work, or recreation?” Individuals could indicate the number of days (Range 1 to 30 days), zero days (coded as “None”), “Don’t know/Not sure” or “Refused”. Like self-rated physical and mental health, we dichotomized self-reported activity limitations coding individuals who indicated that they had zero days of activity limitations or 1 to 13 days of bad physical health as “0” while individuals who had 14 to 30 days of activity limitations as “1”. Individuals who had zero days of poor physical and mental health from the previous two outcomes were also coded as zero. Finally, individuals who indicated “Don’t know/Not sure” or “Refused” were coded as missing.

Sociodemographic Variables/Covariates
These included age, sex, educational attainment, income, and insurance status. These variables were included as they are often confounders of disparities in health outcomes (e.g., age and sex), or serve as social factors that influence health disparities (e.g., educational attainment, income, insurance). Age was coded categorically as “18-39 years old”, “40-64 years old”, and “65+ years old”. Sex was coded dichotomously as “Male” or “Female”. Educational attainment was coded in four categories: “Less than high school”, “Completed high school or

© 2025 McSorley A-MM et al. *JAMA Network Open*.

GED", "Some college or technical school", and "College or above". Individuals with missing educational attainment were coded as missing. Annual income was coded as six categories: "Less than $15,000", "$15,000-$24,999", "$25,000-$34,999", "$35,000-$49,999", "$50,000+", and "Missing". Unlike the other covariates, we include a "Missing" category because of the higher degree of missingness for income variables within surveys. Finally, we included health insurance status as three categories: "Has Insurance", "Does Not Have Insurance", and "Missing". Like income, we included a missing category to due to the higher degree of missingness relative to other variables.

Analytical Sample

When combined, the pooled 2021-2022 BRFSS has total of 883,825 individuals. We first restricted our sample to those who lived in the 50 U.S. States, Guam, Puerto Rico, and the U.S. Virgin Islands (N = 877,390). People who lived in Washington D.C. (N = 6,435) were excluded from the analysis given our analytical question of comparing individuals who live in "U.S. territories" to those who live in formalized U.S. states. We then restrict our sample to those who had complete data on our outcomes of interest and our covariates. Of our 877,390 participants, the number/% missing for each variable was as follows (not mutually exclusive): fair/poor health (n = 2,341; 0.27%), at least 14 days that physical/metal health affected activities (n = 10,010; 1.14%), at least 14 days of poor physical health (n = 20,294; 2.31%), educational attainment (n = 4,819; 0.55%), income (n = 189,215; 21.57%), and insurance (n = 35,035; 3.99%). We created a "Missing" indicator category because of high missingness in the sample overall and to avoid further loss of observations. There was no missingness for age and sex. Excluding people with missing data on our outcomes of interest and covariates (except for income) yielded a final analytical sample of 830,390 (94.64% complete). Our selection of the BRFSS data was to leverage a national data source, with a strong objective protocol that minimizes bias.

Analytical Plan

We first examined the distribution of health outcomes and sociodemographic factors for the full sample and by state/territory. Next, we examined two nested multilevel multivariable logistic models to understand disparities in the four health outcomes by territory compared to the 50 U.S. States (except the Washington D.C.). Our first model examined the age-adjusted odds of the given health outcome by territory. Our second model examined the fully-adjusted association of each health outcome by territory. Our fully adjusted model additionally adjusted for sex, educational attainment, income, health insurance status, and year of survey. Both age-adjusted and fully adjusted models also accounted for the nesting of observations within each state/territory. In this case, Level 1 of the multilevel model is the observation/individual level whereas Level 2 is the state/territory. We calculated Akaike Information Criteria (AIC), Bayesian Information Criteria (BIC) and compared log likelihoods between age-adjusted and fully adjusted models to assess model fit. Afterwards, we converted regression coefficients into predicted prevalence by territory. Our sociodemographic table and prevalences are weighted to account for the complex survey weights used in the BRFSS.

© 2025 McSorley A-MM et al. *JAMA Network Open*.
