# Supplementary material for: Health-Related Quality of Life in the US Territories of Puerto Rico, Guam, and the Virgin Islands
Source: JAMA Netw Open. 2025 Apr 17;8(4):e255646. doi: 10.1001/jamanetworkopen.2025.5646 (PMC12006872; doi:10.1001/jamanetworkopen.2025.5646)
Supplement: Supplement 2. — Data Sharing Statement [file jamanetwopen-e255646-s002.pdf]

Data Sharing Statement

McSorley. Health-Related Quality of Life in the US Territories of Puerto Rico, Guam, and the Virgin Islands. *JAMA Netw Open*. Published April 17, 2025.
doi:10.1001/jamanetworkopen.2025.5646

Data
Data available: Yes
Data types: Other (please specify)
Additional Information: These data are made publicly available on the CDC website.
How to access data: https://www.cdc.gov/brfss/data_documentation/index.htm
When available: With publication

Supporting Documents
Document types: None

Additional Information
Who can access the data: We will share the public link to the data from the CDC
Types of analyses: For any purpose as specified by the CDC
Mechanisms of data availability: Public link:
https://www.cdc.gov/brfss/data_documentation/index.htm
